# Traumatic Dental Injuries in Children and Adolescents from a Major Dental Clinic in Bosnia and Herzegovina: A 5-Year Retrospective Study

**DOI:** 10.3390/medicina60111843

**Published:** 2024-11-08

**Authors:** Olivera Dolic, Marija Obradovic, Zeljka Kojic, Natasa Knezevic, Natasa Trtic, Valentina Veselinovic, Marijana Arapovic-Savic, Mirjana Umicevic-Davidovic, Vanja Krcic

**Affiliations:** Faculty of Medicine, University of Banja Luka, 78 000 Banja Luka, Bosnia and Herzegovina; marija.obradovic@med.unibl.org (M.O.); zeljka.kojic@med.unibl.org (Z.K.); natasa.knezevic@med.unibl.org (N.K.); natasa.trtic@med.unibl.org (N.T.); valentina.veselinovic@med.unibl.org (V.V.); marijana.arapovic-savic@med.unibl.org (M.A.-S.); mirjana.davidovic.umicevic@med.unibl.org (M.U.-D.); vanja.krcic@med.unibl.org (V.K.)

**Keywords:** oral care, traumatic dental injuries, retrospective study, children and adolescents

## Abstract

The aim of this study was to investigate the epidemiology of dental trauma in a public dental clinic in Banja Luka, Bosnia and Herzegovina, from 2019 to 2024. Methods: This research was conducted as a retrospective cross-sectional study. The data were analysed and compared between injured primary and permanent maxillary and mandibular teeth. Results: The review of the dental records revealed 73 patients (49 boys and 24 girls) with TDIs, involving 55 primary and 64 permanent teeth. Most of the patients (27 patients, 36.98%) were aged 7–9 years. The main cause of TDI was falls in both dentitions (81.81% of injured primary teeth and 73.43% of injured permanent teeth). The time of arrival after a TDI for assistance in the dental clinic for most cases was after 24 h in both dentitions, 45.45% of injured primary teeth and 48.43% of permanent teeth. For both dentitions, enamel fractures were the most common injury of hard dental tissues and the pulp, and the necrosis of the maxillary central incisor was the most common complication. Conclusions: It is very important to improve trauma management and increase public knowledge on the way parents seek proper treatment for the TDIs of their children, and in due time.

## 1. Introduction

Traumatic dental injuries (TDIs) are highly prevalent from infancy to adolescence, as 50% of children experience a dental injury before the age of 18 [1]. Although the oral region comprises as small an area as 1% of the total body, dental trauma is almost as high as one-fifth of all bodily injuries [2]. Some studies reported that traumatic dental injuries and their psychosocial consequences may exceed the burden of caries and periodontal disease in the young population [3,4]. Dental trauma can affect quality of life because it alters oral function, appearance, and emotional well-being. A review by Das et al. indicated that a TDI of permanent teeth strongly influences the oral health-related quality of life (OHRQoL) of children and adolescents, and the timely performed dental management of a TDI allows for preventing further biological and socio-psychological impacts [5]. One of the most severe TDIs is avulsion, which can lead to early tooth loss, ankylosis tooth resorption, and irregularities in the growth of the jaws and face due to the infraposition of the teeth [6].

Andreasen reported that the incidence of dental injuries in children is in the range of 1–3% of the population [2]. The prevalence of TDIs in primary and permanent dentitions ranges from 15 to 22%, and dental trauma is more frequent in mixed and permanent dentitions [7]. Injuries to the primary teeth usually occur between 1 and 3 years, and injuries to the permanent teeth from 8 to 12 years [8,9]. The most involved teeth are anterior teeth, with maxillary central and lateral incisors being the most frequently injured teeth [10]. Maxillary central incisors are the most commonly injured teeth in the primary dentition, with a prevalence of 73.9% in the systematic review and meta-analysis by Patnana et al. [11]. Usually, TDI affects a single tooth, but certain types of trauma can affect multiple teeth and are associated with a worse prognosis [9]. The most common causes of dental injuries are falls, participation in sports, and being hit by another person, as well as collisions with various objects. Predisposing factors for dental trauma could be personal characteristics, such as inadequate lip coverage, increased overjet, and class II skeletal malocclusion, the presence of illness, sleep problems, learning difficulties, or physical limitations [10]. A systematic review and meta-analysis by Patnana et al. reported that children with incompetent lip closure were more prone to TDI (49.4%), followed by children with increased overjet, miscellaneous reasons, and children with anterior open bite [11]. The results of studies showed that young boys have a twofold risk of TDI compared to girls at the same age, attributing this to boys being more active and involved in more violent activities and contact sports [10,11,12,13].

In Bosnia and Herzegovina, there are no recent studies, and in the region very few have reported data on TDIs [14,15,16,17,18,19]. Therefore, the aim of our study was to investigate the epidemiology of dental trauma in a public dental clinic in Banja Luka, Bosnia and Herzegovina, from 2019 to 2024.

## 2. Materials and Methods

This retrospective cross-sectional study included data collected from the traumatic dental injury records of paediatric patients (less than 15 years old) who attended the Dental Clinic (Clinic), University of Banjaluka, Bosnia and Herzegovina, between April 2019 and April 2024. The Ethical Committee of the Faculty of Medicine, University of Banja Luka, approved this retrospective cross-sectional study (reference number 18/4.78/22). The Dental Clinic is serviced by a team of dedicated nurses and dentists ranging from dental residents to specialists and professors of paediatric and preventive dentistry. Patients seen by the dental residents were supervised by the same professors and specialists.

The standard procedure of every TDI case in the Clinic includes clinical and radiographic evaluation according to current guidelines [16], then the initial trauma form (dental trauma form—children) for each TDI case is completed. The form consists of patient characteristics (name, age, gender), injury history (time of injury, time of referral to the Clinic, place of injury, aetiology, any symptoms of CNS trauma), comprehensive extra-oral and intra-oral findings, additional information (sensitivity tests, evaluation of tooth colour changes, stage of root development, tooth mobility), radiographic findings, and the treatment provided. Retroalveolar radiographic evaluation is conducted for every TDI case at the baseline and during check-ups. In case of any doubt, cone beam computed tomography (CBCT) is performed to confirm the diagnosis. Every follow-up procedure is subsequently recorded.

For the purpose of the study, information on patients experiencing a TDI was extracted from the dental software of the Clinic. The steps included the selection of the Document section, typing the desired time frame, and then typing the key words, which were “dental trauma form-children”.

The inclusion criteria were as follows:

1. Patients with all types and severities of dental injuries.
2. Patients with complete electronic medical records, including gender, age, visit time, chief complaint, examination, diagnosis, and treatment.

The exclusion criteria were as follows:

1. Patients with incomplete electronic medical records.

The following parameters were recorded: the age and gender of the child, the type of dentition, the type of injured tooth, the main causes of dental injuries, the number of injured teeth per patient, the month of the year the injury occurred, the classification of the injury, trauma complications, and the time interval between the accident and complication.

Following data collection, the first analysis was performed according to age (the first group of children was ≤3 years of age, the second group was 4–6 years of age, the third group was 7–9 years of age, the fourth group was 10–12 years of age, and the fifth group was 13–15 years of age), while the outcome measures were the TDI prevalence and distinguishing between single- or multiple-tooth injuries. Next, measurements were executed in order to distinguish between primary and permanent dentitions where predictor variables were the main causes of dental injuries (being hit by another person, collisions with various objects, and falls), seasons when TDIs happened (autumn, summer, spring, and winter), arrival at the dental clinic (<60 min, 1–2 h, 3–5 h, 6–11 h, 12–23 h, >24 h), and trauma complications (necrosis, colour, pulpitis, periodontitis chronica, and root fracture). TDIs were classified according to traumatic dental injury classifications, Proposal #2130 submitted to the Maintenance Platform of the International Classification of Diseases 11th Revision (ICD-11) in 2018, and the version implemented by the World Health Organisation at the end of the reviewing process in 2022 [20].

The data were analysed using the statistical package for the social sciences’ statistical software program SPSS v22. Descriptive statistics (percentages and mean, SD), the chi-squared test (x^2^), and the Kruskall–Wallis test were used to present and compare the variables. Statistical significance was set at a *p*-value of less than 0.05.

## 3. Results

The review of the dental records revealed 73 patients (49 boys and 24 girls) with TDIs, involving 55 primary and 64 permanent teeth. The mean age of the patients was 6.95 years (SD: 3.65) and the median age was 8 years. The youngest patient was 1.5 years old, and the oldest was 15 years old. Most of the patients (27 patients, 36.98%) were aged 7–9 years, then 0–3 years (20 patients, 27.39%). Among the 73 patients, 40 patients (54.8%) had single-tooth injuries, and 33 patients (45.20%) had multiple-tooth injuries. As much as 60.00% of the children aged 4–6 had multiple-tooth injuries (Table 1).

**Figure 1:**
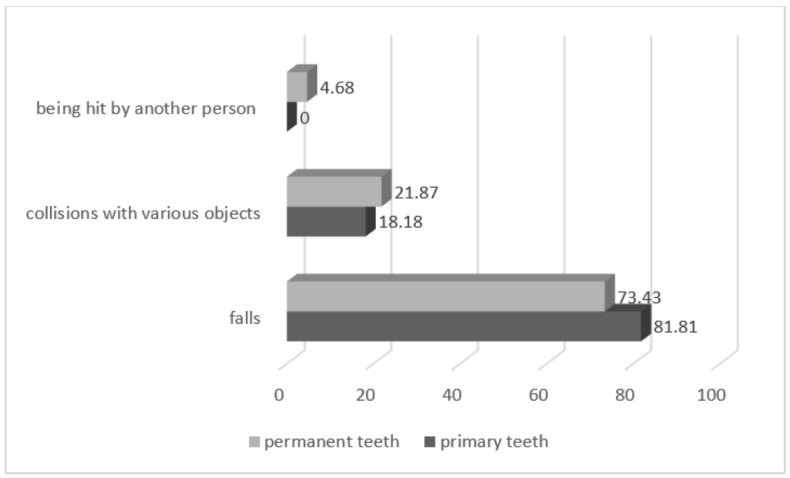
The main causes of TDI (*p* > 0.05).

The main cause of TDI was falls in both dentitions, 81.81% of injured primary teeth and 73.43% of injured permanent teeth (Figure 1). No statistically significant differences were observed in the main cause of TDI between primary and permanent teeth (*p* > 0.05).

**Figure 2:**
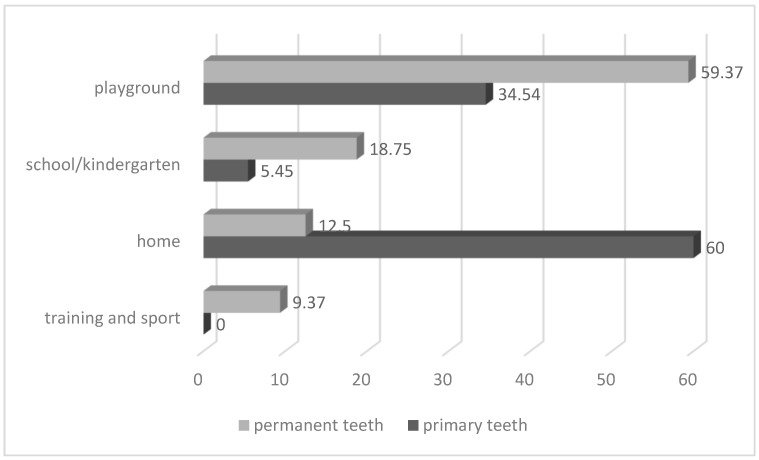
Place where TDI occurred (*p* < 0.00001).

Most accidents involving primary teeth occurred at home (60% of injured primary teeth) and at playgrounds (34.54% of injured primary teeth) (Figure 2). Most accidents involving permanent teeth occurred at playgrounds (59.37% of injured permanent teeth) and at school (18.75% of injured permanent teeth) (Figure 2). Comparisons of TDIs between primary and permanent teeth showed statistically significant differences in the place where the TDI occurred (*p* < 0.00001).

**Figure 3:**
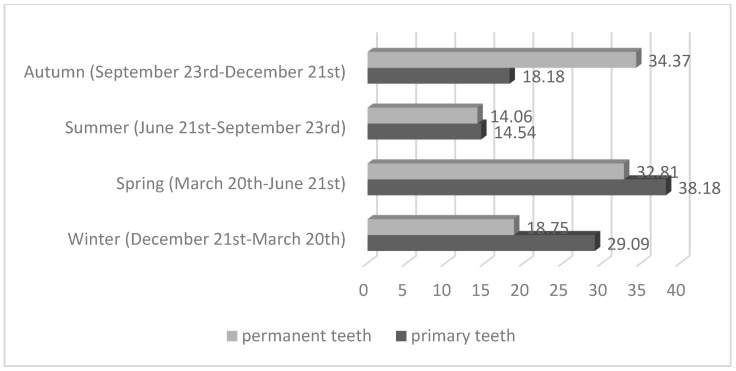
Month of year when TDI occurred (*p* > 0.05).

Injuries of primary teeth mostly occurred in spring (38.18%), then in winter (29.09%), and injuries of permanent teeth mostly happened in autumn (34.75%) and in spring (32.81%) (Figure 3). No statistically significant differences were observed between primary and permanent teeth.

The arrival time for most TDI cases was the next day (after 24 h) in both dentitions, 45.45% of injured primary teeth and 48.43% of permanent teeth (Table 2). Comparisons between primary and permanent teeth showed no statistically significant differences (*p* > 0.05).

The distribution of teeth with different trauma types for the permanent and primary dentitions and the statistical significance are presented in Table 3. For the primary dentition, enamel fracture was the most common injury of hard dental tissues and the pulp (36.36%), followed by enamel dentin pulp fracture (27.27%). For the permanent dentition, enamel fractures were also the most common injury of hard dental tissues and the pulp (35.89%), followed by enamel dentin fractures (28.2%). No statistically significant differences were observed in the prevalence of the injuries of hard dental tissues and the pulp between primary and permanent teeth (*p* > 0.05). For both dentitions, lateral luxation was the most common injury of periodontal tissues (20.45% for primary and 24.00% for permanent). No statistically significant differences were observed in the prevalence of the injuries of periodontal tissues between primary and permanent teeth (*p* > 0.05). Maxillary central incisors in both dentitions were the most frequently traumatised, followed by maxillary lateral incisors, while the mandibular lateral incisor was the least traumatised tooth in both dentitions.

For both dentitions, the necrosis of the maxillary central incisor was the most common complication (75.00% for primary and 65.00% for permanent). The time of occurrence of the complication was less than 10 days since the TDI happened. Comparisons of TDIs between primary and permanent teeth showed no statistically significant differences according to the type of complication and the time of occurrence of the complication (*p* > 0.05) (Table 4).

## 4. Discussion

Traumatic dental injury can result in pain, a loss of function, poor aesthetics, and psychological trauma and represents a serious problem associated with many aspects of the child’s life. TDI has shown differences in various parts of the world, so it is important to see global parallels as well as local differences. The most commonly investigated parameters are the age-wise distribution of trauma, frequency, aetiology, the classification of injuries, trauma complications, and the time interval between the accident and the complication.

The results of the present study showed that children aged between 7 and 9 years are more likely to suffer a TDI. Our results are similar to those of Sandalli, N. et al., as well as Díaz, J. A. et al. and Oldin, A. et al., who also found the highest incidence of dental trauma at the age of 8 years [21,22,23]. Studies carried out by Bastone, E. B. et al., Ozge Eyuboglu et al., and Beera, S. et al. showed that the highest prevalence of TDIs was recorded in the age group of 2–6-year-old children, followed by children aged 7–12 and 13–18 years [24,25,26]. A systematic review and meta-analysis by Patnana et al. reported that, from the included studies, falls contribute to the highest number (59.3%) of TDIs in primary teeth. This is explained by the fact that children in the age group of 2–5 years learn to walk and run while developing their motor coordination skills [11]. Agouropoulos, A. et al. have reported that age groups of 7–11 years have the highest odds of experiencing a dental injury [10]. Lam et al. concluded that older age groups (10−14 years) were correlated with the highest trauma frequency [27]. Other studies showed that teenagers older than 14 years are more likely to suffer a traumatic injury [25,28]. It can be concluded that age is a risk factor, related to the region as well as the different activities and habits of children.

Regarding the number of injured teeth in the present study, 54.80% of patients had single-tooth injuries, which was similar to the study of Agouropoulos, A. et al., who found 48% of patients had trauma to a single tooth [10]. Studies by Noori, A. J. et al. and Artun, J. et al. showed that even more patients had only one injured tooth, 69.5% and 77.3%, respectively [29,30]. Shayegan, A. et al. reported that the majority of dental injuries affected two teeth (51%) [31]. In the study of Guedes, O.A. et al., in most patients (81.75%) TDI occurred in more than one tooth [32].

In the present study, 81.81% of injured primary teeth and 73.43% of injured permanent teeth were caused by falls. These rates are higher when compared to results from Alhaddad, B. et al. (17.62%), Rouhani, A. et al. (42.9%), Chopra, A. et al. (51.14%), Nagarajappa, R. et al. (52.5%), and Abdel Malak, C. et al. (52.5%) [33,34,35,36,37]. Beera, S. reported that the majority of TDIs had occurred because of an accident or by falling (92.6%), as well as studies carried out by Reddy, K.V. et al., Juneja, P. et al., and Tewari, N. et al. [26,38,39,40].

The findings of the present study reveal that 60% of cases in primary teeth occurred at home. Our results are the same as those recorded by Galea et al. (60.00% of cases) and similar to the results of Alhaddad, B et al., as dental injuries at home comprised 46.47% of cases [33,41]. In comparison to the observations by Lembacher, S. et al. (79.4%), the results of our survey are much lower [42]. This is expected due to the fact that little children spend most of the time at home. In a familiar setting, they can be left unattended more often. They are more prone to falls because of their developmental stage and lack of motor coordination when they learn to walk or play. Also, the data were collected during COVID-19 and the pandemic-associated social restriction might have contributed to this. Accidents at playgrounds (59.37%) were the major source of injury in the permanent dentition in the present study. There are some variations between studies and countries regarding places where permanent teeth injuries happened, so the review of Bastone et al. concluded that accidents at home and school accounted for most injuries to the permanent dentition [24].

The relationship between seasons and dental trauma in the present study showed an increase in TDIs in spring (38.18% of cases for primary and 32.81% of cases for permanent teeth) and in autumn (34.75% of cases for permanent teeth). Spring is the season when the weather is pleasant and outdoor activities are more common among children, increasing the probability of suffering a TDI. Alhaddad et al. reported that 34.14% of children were affected by traumatic injuries during spring in their study [33]. Other studies also reported the same [10,43,44], but in the study by Sælen et al., there was no clear seasonal trend regarding when patients experienced TDIs [45].

Concerning the hospital arrival time following a TDI, 45.45% of patients for primary teeth and 48.43% of patients for permanent teeth sought treatment within between 24 h and 48 h in the present study. Our results are similar to those recorded by Özgür et al., who reported that 46.3% of patients with traumatised primary teeth sought treatment after more than 24 h [46]. Odersjo, M.L. et al. and Zaitoun, H. et al. have suggested that the most common reasons for late referral are the underestimation of primary teeth trauma by the parents and a prolonged transit time or parental unavailability [47,48]. While the findings for primary teeth can be somewhat understood, it is inconceivable, according to our results, that the parents of children with injuries to permanent teeth delayed treatment. Unfortunately, this is a confirmation of unfamiliarity with the necessity of treatment. Other researchers found shorter response times. Lembacher, S. et al. reported that 66.0% of patients with traumatised permanent teeth sought treatment within the first 24 h after a TDI with a maximum response time of 2 h [42]. In another study, Guo et al. found that 74.8% of patients presented themselves to the emergency department within 4 h, and more than 90% presented themselves within 12 h, and they assumed that the reason was that trauma is more likely to attract patients’ attention than other dental diseases [49].

The maxillary central incisors were the most frequently injured teeth in both dentitions. Many studies have also reported that the majority of trauma cases involve the anterior teeth, predominantly the maxillary central incisors [2,9,33,40,42,45,50]. The reason for these results is clear due to the maxillary central incisors’ frontal and most prominent position in the oral cavity. Tewari, N. et al., in their review and meta-analyses, reported that inadequate lip coverage, tooth protrusion, and convex profiles were regarded as the primary risk factors for TDI and this could be the reason for the injury of maxillary central incisors being most frequent [40]. In the present study, no such data were recorded, so we could not analyse a possible correlation between TDIs and orthodontic anomalies.

The type of dental injury should be considered with caution due to differences in classification systems across the globe. In the present study, enamel fractures were the most common injuries of hard dental tissues and the pulp for primary and permanent teeth, 36.36% and 35.89%, respectively. Consistent with the findings of this study, no matter the classification system, uncomplicated crown fractures without pulp involvement have been reported as the most common type of injury in most of the literature [4,9,10,24,36,42,45,51].

When evaluating injuries of periodontal tissue, lateral luxation was the most common (20.45% for primary and 24.00% for permanent). de Paula Barros et al. reported that lateral luxation in 42.00% of cases is the most common in the permanent dentition, but for primary teeth, subluxation (27.20%) was the most common [9]. The three most prevalent types of injury of periodontal tissues in the permanent dentition in the study by Lembacher et al. were concussions (21.7%), subluxations (27.5%), and lateral dislocations (26.7%) [42]. One-third of the luxation injuries in the study by Agouropoulos et al. were avulsions, and this frequency is quite high compared to the results of our and similar reports [10].

Only four (7.27%) injured primary teeth had some trauma complications in the present study. The most frequent complications were pulp necrosis, occurring in 75% of teeth with a complication. Unfortunately, 20 (31.25%) injured permanent teeth had complications, and 70% of complications were pulp necrosis. Concerning the time of occurrence of the complication, 75.0% of primary teeth and 70% of permanent teeth had complications within less than 10 days after the TDI happened. The delay in obtaining emergency care could be one of the reasons for the results of complications. Some studies regarded that after-hours injuries were more likely to result in complications [52,53]. Antipovienė, A. et al. reported similar results, and they showed that complications related to pulp necrosis (pulp necrosis, periapical periodontitis, and abscess formation) were the most frequent complications in the primary (92%) and permanent (54%) dentition, but more complications occurred within less than 3 months after the TDI [54].

Simultaneous different types of injuries occurring on the same tooth are more harmful than a single injury [55]. A higher incidence of pulp necrosis and infection is seen in fractured teeth, with or without pulp exposure, along with luxation injuries [6]. Amilcar et al. reported that patients with severe TDIs, such as luxation with or without tooth fracture, typically seek emergency care [56]. They highlight the importance of understanding the clinical and demographic profiles of these injuries to develop comprehensive short-term and long-term treatment strategies [56].

Numerous studies showed that TDIs represent a significant health issue that may be influenced by socioeconomic conditions [57,58,59,60,61,62,63]. Mira et al. reported that adolescents whose parents were always unemployed, as well as families that never owned a car, had greater odds of having TDIs than those whose parents were never unemployed and had a family car [61]. Bezerra et al. assessed the relationship between contextual (place of residence and socioeconomic indicators) and individual characteristics, including sex, family income, parents/guardians’ years of schooling, overjet and open bite, self-esteem, sense of coherence, oral health beliefs, social support, and TDIs in 12-year-old schoolchildren in a 2-year follow-up [63]. The authors in this study asserted the association of low self-esteem and a low sense of coherence with a greater likelihood of TDIs. A study by Bratteberg et al. revealed that TDIs were more frequent among adolescents with adverse psychosocial scores. The same authors regarded that psychosocial factors may evince a protective effect against TDIs through behaviours, including the more frequent use of safety equipment in games and sports practice, as well as by avoiding risky activities for TDIs [60]. Bernandino et al. reported that the prevalence of TDIs was higher among children who used electronic devices and whose screen time was more than 2 h per day, as well as among the children of single parents, those whose families were classified as chaotic, those who studied at public schools, and those whose schools had a rigid courtyard floor (cement/ceramic/granite) [64]. Power relations among family members can exert an influence on the needs of children. Chaotic families may predispose children to TDI because they have greater flexibility, no established family leader, a frequent changing of rules, and less supervision of daily activities [64]. 

The current study has not involved the impact of background factors such as income, education, employment, social status, physical activity level, or parental supervision practices; therefore, new research is essential to fully understand how these factors contribute to TDI in children and adolescents. The findings of this study have some implications for policy and research and highlight the importance of health promotion interventions. Achieving effective patient education in dental traumatology involves a comprehensive approach. Efforts should be made to educate parents, children, teachers, trainers, coaches, and other individuals who care about children on accident prevention and safe play practices to instil early habits that promote oral health and reduce the risk of injuries. Targeted educational campaigns can emphasise the importance of dental safety during everyday life, recreational activities, and sports. In campaigns utilising digital apps, visual aids, games, interactive workshops, and educational sessions, it is possible to reinforce preventive measures and continuous updates, follow up, and promote patient empowerment. There is substantial evidence supporting the effectiveness of mouthguards in preventing or reducing the severity of TDIs [65]. Dentists and other healthcare providers should play an active role by providing regular education and anticipatory guidance regarding the importance of using mouthguards and helmets in sports and all activities where that is necessary. Protrusive teeth have been found to place a child at a higher risk of TDIs, so it is important to involve orthodontic correction of the overjet and/or the use of protective equipment. Having a readily accessible trauma kit can help reduce stress for the dental team and the patient, enhancing the likelihood of a favourable outcome [19]. A lack of capability (knowledge or skill), opportunity (access, resources), or motivation (desire, awareness) can hinder adherence to preventive methods, so it is important to continue educating dentists about preventive and treatment measures according to updated trauma management guidelines for TDI. Crucial in providing appropriate care is the possession of the necessary materials and expertise to adhere to guidelines for TDI treatment by dentists. 

TDI prevention and treatment requires a comprehensive team approach to ensure holistic and effective patient care. The Board of Directors of the International Association of Dental Traumatology (IADT) and the Academy for Sports Dentistry (ASD) reviewed and approved the Guidelines for Prevention of Traumatic Dental Injuries, and they are available at their link or in the form of articles [66,67]. The therapy of tooth injuries depends on the type of injury and the type of tooth that is injured (primary or permanent). For that reason, the world’s leading web-based tool in dental traumatology, the Dental Trauma Guide, was developed and managed in cooperation with the University Hospital of Copenhagen, Denmark, and the International Association of Dental Traumatology (IADT) [68]. This guide is an evidence-based treatment guide and has been constantly updated based on the research of the Dental Trauma Guide team.

### Limitations of Study

The limitations of our study, as well as any retrospective research, are that the presented data were already collected, and it was not possible to include certain variables that may be significant for the therapeutic outcome of TDI. This can introduce recall bias, where critical details about injuries or treatments may not have been accurately recorded or recalled by patients and caregivers. Conducting a prospective study in the future would allow for real-time data collection, enhancing the accuracy and reliability of the data. Additionally, the examinees of this research come from one region and one clinic, and the number of TDIs is small. If data were collected from several centres and regions, it could increase generalizability, and there might be more statistical significance, or another important variable in the epidemiology of TDI could be singled out. On the other hand, since the Dental Clinic is a part of the Faculty of Medicine, Department of Dentistry, including undergraduate and postgraduate study, the management of trauma implies a detailed clinical and radiographic examination and better diagnosis, something that maybe is not possible in large-scale epidemiological studies.

## 5. Conclusions

This study has shown a seasonal peak of TDIs in spring. Falls were the leading etiological factor. The maxillary incisors were more often involved in trauma, and a long time interval between the accident and treatment was observed (between 24 h and 48 h). Based on the results of the present study, it is very important to increase public health knowledge and parental awareness regarding the emergency management of dental trauma in children.

Conducting a prospective study in the future including background factors would allow for a better understanding of the relationship between exposure factors and outcomes of TDI in children and adolescents.

## Figures and Tables

**Table 1:** Distribution according to age and multiple injured teeth.

Age Groups	Patients with TDI		Patients with Multiple Injured Teeth	
	*n*	%	*n*	%
0–3	20	27.39	11	55.00
4–6	10	13.69	6	60.00
7–9	27	36.98	12	44.44
10–12	10	13.69	3	30.00
13–15	6	8.21	1	16.66
Σ	73	100	33	45.20

*n*, number of patients.

**Table 2:** The time interval between a TDI and arrival at the dental clinic (*p* > 0.05).

Time Interval	Primary Teeth		Permanent Teeth	
	*n*	%	*n*	%
<60 min	0	0	3	4.68
1–2 h	0	0	0	0
3–5 h	0	0	0	0
6–11 h	0	0	0	0
12–23 h	0	0	0	0
>24 h	25	45.45	31	48.43
2–10 days	20	36.36	14	21.87
>10 days	10	18.18	16	25
Σ	73		100	

*n*, number of teeth; *p* > 0.05.

**Table 3:** Prevalence (%) of different types of TDI and statistical difference between primary and permanent dentitions.

	Primary Dentition									Permanent Dentition									
	Maxilla				Mandibula					Maxilla				Mandibula					
	Central Incisor		Lateral Incisor		Central Incisor		Lateral Incisor			Central Incisor		Lateral Incisor		Central Incisor		Lateral Incisor			
	*n*	%	*n*	%	*n*	%	*n*	%	Σ	*n*	%	*n*	%	*n*	%	*n*	%	Σ	*p*
Injury of hard dental tissues and the pulp																			
Enamel infraction	0	0	0	0	0	0	0	0	0	5	12.82	2	5.12	0	0	0	0	7	0.2001
Enamel fracture	4	36.36	2	18.18	0	0	0	0	6	14	35.89	1	2.56	0	0	0	0	15	
Enamel dentin fracture	0	0	0	0	0	0	0	0	0	11	28.20	0	0	1	2.56	0	0	12	
Enamel dentin pulp fracture	3	27.27	0	0	0	0	0	0	3	2	5.12	0	0	2	5.12	1	2.6	5	
Root fracture	2	18.18	0	0	0	0	0	0	2	0	0	0	0	0	0	0	0	0	
Crown root fracture	0	0	0	0	0	0	0	0	0	0	0	0	0	0	0	0	0	0	
Σ	9	81.81	2	18.18	0	0	0	0	11	32	82.05	3	7.69	3	7.69	1	2.6	39	
Injury of periodontal tissues																			
Concussion	3	6.81	2	4.54	0	0	0	0	5	1	4.00	2	8.00	2	8.00	1	4.00	6	0.2297
Subluxation	1	2.27	2	4.54	1	2.27	0	0	4	5	20.00	0	0	1	4.00	0	0	6	
Intrusive luxation	5	11.36	2	4.54	1	2.27	2	4.5	10	4	16.00	1	4.00	0	0	0	0	5	
Extrusive luxation	5	11.36	1	2.27	0	0	0	0	6	1	4.00	0	0	0	0	0	0	1	
Lateral luxation	9	20.45	3	6.81	1	2.27	0	0	13	6	24.00	0	0	0	0	0	0	6	
Avulsion	5	11.36	1	2.27	0	0	0	0	6	1	4.00	0	0	0	0	0	0	1	
Σ	28	63.63	11	25	3	6.81	2	4.5	44	18	72.00	3	12.00	3	12.00	1	4.00	25	

*n*, number of teeth; *p*-value, statistical significance. Note: the same tooth could have more than one condition.

**Table 4:** Prevalence (%) of different types of complication, the time of occurrence of the complication, and the statistical difference between primary and permanent dentitions.

	Primary Dentition									Permanent Dentition									
	Maxilla				Mandibula					Maxilla				Mandibula					
	Central Incisor		Lateral Incisor		Central Incisor		Lateral Incisor			Central Incisor		Lateral Incisor		Central Incisor		Lateral Incisor			
	*n*	%	*n*	%	*n*	%	*n*	%	Σ	*n*	%	*n*	%	*n*	%	*n*	%	Σ	*p*
Type of complication																			
Necrosis	3	75.00	0	0	0	0	0	0	3	13	65.00	1	5.00	1	5.00	0	0	15	0.1436
Colour	1	25.00	0	0	0	0	0	0	1	2	10.00	0	0	0	0	0	0	2	
Pulpitis	0	0	0	0	0	0	0	0	0	1	5.00	0	0	0	0	0	0	1	
Periodontitis chronica	0	0	0	0	0	0	0	0	0	1	5.00	0	0	0	0	0	0	1	
Root fracture	0	0	0	0	0	0	0	0	0	1	5.00	0	0	0	0	0	0	1	
Σ	4	100.00	0		0		0		4	18	90.00	1	5.00	1	5.00	0		20	
The time of occurrence of the complication																			
<10 days	3	75	0	0	0	0	0	0	3	13	65.00	1	5.00	1	5.00	0	0	15	0.21
10–30 days	1	25	0	0	0	0	0	0	1	2	10.00	0	0	0	0	0	0	2	
1–6 months	0	0	0	0	0	0	0	0	0	1	5.00	0	0	0	0	0	0	1	
9–12 months	0	0	0	0	0	0	0	0	0	1	5.00	0	0	0	0	0	0	1	
>12 months	0	0	0	0	0	0	0	0	0	1	5.00	0	0	0	0	0	0	1	
Σ	4	100.00	0		0		0		4	18	90.00	1	5.00	1	5.00	0		20	

*n*, number of teeth; *p*-value, statistical significance. Note: the same tooth could have more than one condition.

## Data Availability

Data will be available upon request to the corresponding author.

## References

[1] Ritwik P., Massey C., Hagan J. (2015). Epidemiology and outcomes of dental trauma cases from an urban pediatric emergency department. Dent. Traumatol..

[2] Andersson L. (2013). Epidemiology of traumatic dental injuries. J. Endod..

[3] Flores M.T., Andersson L., Andreasen J.O., Bakland L.K., Malmgren B., Barnett F., Bourguignon C., DiAngelis A., Hicks L., Sigurdsson A. (2007). Guidelines forthe management of traumatic dental injuries. I. Fractures andluxations of permanent teeth. Dent. Traumatol..

[4] Lam R. (2016). Epidemiology and outcomes of traumatic dental injuries: A review of the literature. Aust. Dent. J..

[5] Das P., Mishra L., Jena D., Govind S., Panda S., Lapinska B. (2022). Oral health-related quality of life in children and adolescents with a traumatic injury of permanent teeth and the impact on their families: A systematic review. Int. J. Environ. Res. Public Health.

[6] Budak L., Levin L. (2024). The Importance of Immediate Dental Trauma Care: Comprehensive Education, Treatment Approaches, and Their Profound Impact on Patients’ Quality of Life. Dent. Traumatol..

[7] Petti S., Glendor U., Andersson L. (2018). World traumatic dental injury prevalence and incidence, a meta-analysis-Onebillion living people have had traumatic dental injuries. Dent. Traumatol..

[8] Schuch H.S., Goettems M.L., Correa M.B., Torriani D.D., Demarco F.F. (2013). Prevalence and treatment demand after traumatic dental injury in South Brazilian schoolchildren. Dent. Traumatol..

[9] de Paula Barros J.N., de Araújo T.A.A., Soares T.R.C., Lenzi M.M., de Andrade Risso P., Fidalgo T.K.D.S., Maia L.C. (2019). Profiles of Trauma in Primary andPermanent Teeth of Children and Adolescents. J. Clin. Pediatr. Dent..

[10] Agouropoulos A., Pavlou N., Kotsanti M., Gourtsogianni S., Tzanetakis G., Gizani S. (2021). A 5-year data report of traumatic dental injuries in children and adolescents from a major dental trauma center in Greece. Dent. Traumatol..

[11] Patnana A.K., Chugh A., Chugh V.K., Kumar P., Vanga N.R.V., Singh S. (2021). The prevalence of traumatic dental injuries in primary teeth: A systematic review and meta-analysis. Dent. Traumatol..

[12] Skaare A.B., Jacobsen I. (2003). Dental injuries in Norwegians aged 7–18 years. Dent. Traumatol..

[13] Tello G., Bonini G.C., Murakami C., Abanto J., Oliveira L.B., Bönecker M. (2016). Trends in the prevalence of traumatic crown injuries and associated factors in Brazilian preschool children: 10-year observational data. Dent. Traumatol..

[14] Vuković A., Marković D., Petrović B., Apostolović M., Golijanin R., Kanjevac T., Stojković B., Perić T., Blagojević D. (2013). Traumatic dental injuries in Serbian children--epidemiological study. Srp. Arh. Celok. Lek..

[15] Vuletić M., Škaričić J., Batinjan G., Trampuš Z., Bagić I.Č., Jurić H. (2014). A retrospective study on traumatic dental and soft-tissue injuries in preschool children in Zagreb, Croatia. Bosn. J. Basic Med. Sci..

[16] Škaričić J., Vuletić M., Hrvatin S., Jeličić J., Čuković-Bagić I., Jurić H. (2016). Prevalence, type and etiology of dental and soft tissue injuries in children in Croatia. Acta Clin. Croat..

[17] Igić A., Stojković B., Mitić A., Acović A., Todorović K., Todorović A., Igić M. (2021). Injuries to the primary teeth and soft tissues in children in Nis, Serbia. Acta Medica Median..

[18] Nashkova S., Dimova C. (2022). Traumatic dental injuries: Etiology, prevalence and possible outcomes. MEDIS—Int. J. Med. Sci. Res..

[19] Šimunović L., Špiljak B., Vranić L., Negovetić Vranić D. (2024). Treatment priorities and arrival time of traumatic dental injuries—An 8-year retrospective study. Dent. Traumatol..

[20] World Health Organization Team Classifications and Terminologies (2024). NA0D Injury of Teeth or Supporting Structures.

[21] Sandalli N., Cildir S., Guler N. (2005). Clinical investigation of traumatic injuries in Yeditepe University, Turkey during the last 3 years. Dent. Traumatol..

[22] Díaz J.A., Bustos L., Brandt A.C., Fernández B.E. (2010). Dental injuries among children and adolescents aged 1–15 years attending to public hospital in Temuco, Chile. Dent. Traumatol..

[23] Oldin A., Lundgren J., Nilsson M., Norén J.G., Robertson A. (2015). Traumatic dental injuries among children aged 0–17 years in the BITA study-a longitudinal Swedish multicenter study. Dent. Traumatol..

[24] Bastone E.B., Freer T.J., McNamara J.R. (2000). Epidemiology of dental trauma: A review of the literature. Aust. Dent. J..

[25] Eyuboglu O., Yilmaz Y., Zehir C., Sahin H. (2009). A 6-year investigation into types of dental trauma treated in a paediatric dentistry clinic in Eastern Anatolia region, Turkey. Dent. Traumatol..

[26] Beera S. (2021). Prevalence of Traumatic Dental Injuries and Their Correlation with Associated Factors in Children and Adolescents. Doctoral Dissertation.

[27] Lam R., Abbott P., Lloyd C., Lloyd C., Kruger E., Tennant M. (2008). Dental trauma in an Australian rural center. Dent. Traumatol..

[28] Lauridsen E., Hermann N.V., Gerds T.A., Kreiborg S., Andreasen J.O. (2012). Pattern of traumatic dental injuries in the permanent dentition among children, adolescents, and adults. Dent. Traumatol..

[29] Noori A.J., Al-Obaidi W.A. (2009). Traumatic dental injuries among primary school children in Sulaimani city, Iraq. Dent. Traumatol..

[30] Artun J., Behbehani F., Al-Jame B., Kerosuo H. (2005). Incisor trauma in an adolescent Arab population: Prevalence, severity, and occlusal risk factors. Am. J. Orthod. Dentofac. Orthop..

[31] Shayegan A., De Maertelaer V., Vanden Abbeele A. (2007). The prevalence of traumatic dental injuries: A 24-month survey. J. Dent. Child..

[32] Guedes O.A., de Alencar A.H., Lopes L.G., Pécora J.D., Estrela C. (2010). A retrospective study of traumatic dental injuries in a Brazilian dental urgency service. Braz. Dent. J..

[33] Alhaddad B., Rozsa N.K., Tarjan I. (2019). Dental trauma in children in Budapest. A retrospective study. Eur. J. Paediatr. Dent..

[34] Rouhani A., Movahhed T., Ghoddusi J., Mohiti Y., Banihashemi E., Akbari M. (2015). Anterior traumatic dental injuries in East Iranian school children: Prevalence and risk factors. Iran. Endod. J..

[35] Chopra A., Lakhanpal M., Rao N., Gupta N., Vashisth S. (2014). Traumatic dental injuries among 12–15-year-old-school children in Panchkula. Arch. Trauma Res..

[36] Nagarajappa R., Ramesh G., Uthappa R., Kannan S.P.K., Shaikh S. (2020). Risk factors and patterns of traumatic dental injuries among Indian adolescents. J. Dent. Sci..

[37] Abdel Malak C., Chakar C., Romanos A., Rachidi S. (2021). Prevalence and etiological factors of dental trauma among 12-and 15-year-old schoolchildren of Lebanon: A national study. Sci. World J..

[38] Reddy K.V., Kumar K.N., Venkatasubramanian R., Togaru H., Kannakiah S., Reddy R. (2017). Incidence of traumatic dental injuries in children aged 3–18 years in Tirupathi. Int. J. Pedod. Rehabil..

[39] Juneja P., Kulkarni S., Raje S. (2018). Prevalence of traumatic dental injuries and their relation with predisposing factors among 8-15 years old school children of Indore city, India. Clujul Med..

[40] Tewari N., Mathur V.P., Siddiqui I., Morankar R., Verma A.R., Pandey R.M. (2020). Prevalence of traumatic dental injuries in India: A systematic review and metaanalysis. Indian J. Dent. Res..

[41] Galea H. (1984). An investigation of dental injuries treated in an acute care general hospital. J. Am. Dent. Assoc..

[42] Lembacher S., Schneider S., Lettner S., Bekes K. (2022). Prevalence and Patterns of Traumatic Dental Injuries in the Permanent Dentition: A Three-Year Retrospective Overview Study at the University Dental Clinic of Vienna. Int. J. Environ. Res. Public Health.

[43] Joachim M., Tuizer M., Araidy S., Abu E.-N. (2018). Pediatric maxillofacial trauma: Epidemiologic study between the years 2012 and 2015 in an Israeli medical center. Dent. Traumatol..

[44] Kırzıoglu Z., Oz E. (2019). Changes in the aetiological factors of dental trauma in children over time: An 18-year retrospective study. Dent. Traumatol..

[45] Sælen F.D., Virtanen J.I., Skeie M.S., Sulo G., Thelen D.S. (2024). Traumatic dental injuries among children attending the public after-hours emergency dental clinic in Bergen, Norway. Acta Odontol. Scand..

[46] Özgür B., Ünverdi G.E., Güngör H.C., McTigue D.J., Casamassimo P.S. (2021). A 3-Year retrospective study of traumatic dental Injuries to the primary dentition. Dent. Traumatol..

[47] Odersjo M.L., Robertson A., Koch G. (2018). Incidence of dental traumatic injuries in children 0–4 years of age: A prospective study based on parental reporting. Eur. Arch. Paediatr. Dent..

[48] Zaitoun H., North S., Lee S., Albadri S., McDonnell S.T., Rodd H.D. (2010). Initial management of paediatric dento-alveolar trauma in the permanent dentition: A multi-centre evaluation. Br. Dent. J..

[49] Guo H.Q., Yang X., Wang X.T., Li S., Ji A.P., Bai J. (2021). Epidemiology of maxillofacial soft tissue injuries in an oral emergency department in Beijing: A two-year retrospective study. Dent. Traumatol..

[50] Faus-Matoses V., Faus-Matoses I., Ruiz-Sanchez C., Faus-Damia M., Faus-Llacer V.J. (2020). Incidence of traumatic dental injury in Valencia, Spain. Med. Oral Patol. Oral Cir. Buccal.

[51] Costantinides F., Tonizzo M., Dotto F., Lenhardt M., Borella A., Sclabas M., Rizzo R., Maglione M. (2023). Epidemiological aspects of dental trauma associated with maxillofacial injures: Ten years of clinical experience in Trieste, Italy. Dent. Traumatol..

[52] Lima T.F.R., Silva E.J.N.L.D., Gomes B.P.F.A., Almeida J.F.A., Zaia A.A., Soares A.J. (2017). Relationship between initial attendance after dental trauma and development of external inflammatory root resorption. Braz. Dent. J..

[53] Kallel I., Douki N., Amaidi S., Ben A.F. (2020). The incidence of complications of dental trauma and associated factors: A retrospective study. Int. J. Dent..

[54] Antipovienė A., Narbutaitė J., Virtanen J.I. (2021). Traumatic dental injuries, treatment, and complications in children and adolescents: A register-based study. Eur. J. Dent..

[55] Bourguignon C., Cohenca N., Lauridsen E., Flores M.T., O’Connell A.C., Day P.F., Tsilingaridis G., Abbott P.V., Fouad A.F., Hicks L. (2020). International Association of Dental Traumatology Guidelines for the Management of Traumatic Dental Injuries: 1. Fractures and Luxations. Dent. Traumatol..

[56] Amilcar A.L.L., Vieira W.A., Matta A.C.G., de Almeida Gomes B.P.F., da Silva M.A.M., de Almeida J.F.A., Ferraz C.C.R., Santos E.C.A., Neto J.V., Soares A.d.J. (2024). Epidemiological Profile of Luxations Injuries With or Without Dental Fractures in Permanent Teeth: A 10-Years Retrospective Study. Dent. Traumatol..

[57] Corrêa-Faria P., Martins C.C., Bönecker M., Paiva S.M., Ramos-Jorge M.L., Pordeus I.A. (2015). Absence of an association between socioeconomic indicators and traumatic dental injury: A systematic review and meta-analysis. Dent. Traumatol..

[58] Blokland A., Watt R.G., Tsakos G., Heilmann A. (2016). Traumatic dental injuries and socioeconomic position—Findings from the Children’s Dental Health Survey 2013. Community Dent. Oral Epidemiol..

[59] Sideri S., Marcenes W., Stansfeld S.A., Bernabé E. (2018). Family environment and traumatic dental injuries in adolescents. Dent. Traumatol..

[60] Bratteberg M., Thelen D.S., Klock K.S., Bardsen A. (2019). Traumatic dental injuries and experiences along the life course—A study among 16-yr-old pupils in western Norway. Eur. J. Oral Sci..

[61] Magno M.B., Nadelman P., Leite K.L.F., Ferreira D.M., Pithon M.M., Maia L.C. (2020). Associations and risk factors for dental trauma: A systematic review of systematic reviews. Community Dent. Oral Epidemiol..

[62] Mira R.S., Marcenes W., Stansfeld S.A., Bernabé E. (2021). Cumulative socio-economic disadvantage and traumatic dental injuries during adolescence. Dent. Traumatol..

[63] Bezerra E.D.F.N., Herkrath F.J., Vettore M.V., Rebelo M.A.B., de Queiroz A.C., Rebelo Vieira J.M., Pereira J.V., Freitas M.O.d.S., Herkrath A.P.C.d.Q. (2024). Contextual and individual factors associated with traumatic dental injuries in deprived 12-year-old schoolchildren: A cohort study. Dent. Traumatol..

[64] Bernardino V.M.M., De Lima L.C.M., Neves É.T.B., De Paiva S.M., Granville-Garcia A.F. (2024). Individual and contextual determinants associated with traumatic dental injuries in children eight to ten years of age: A multilevel analysis. Acta Odontol. Scand..

[65] Fernandes L.M., Neto J.C.L., Lima T.F.R., Magno M.B., Santiago B.M., Cavalcanti Y.W., Almeida L.d.F.D.d. (2018). The use of mouthguards and prevalence of dento-alveolar trauma among athletes: A systematic review and meta-analysis. Dent. Traumatol..

[66] International Association of Dental Traumatology (IADT), Academy for Sports Dentistry (ASD) (2020). IADT-ASD Guidelines for the Prevention of Traumatic Dental Injuries. https://iadt-dentaltrauma.org/guidelines-and-resources/iadt-asd-guidelines/.

[67] IADT-ASD Guidelines for the Prevention of Traumatic Dental Injuries (2024). The International Association of Dental Traumatology (IADT) and the Academy for Sports Dentistry (ASD) guidelines for prevention of traumatic dental injuries: Part 1–10. Dent. Traumatol..

[68] Dental Trauma Guide (Internet). https://dentaltraumaguide.org/free-version/?r=250&wcm_redirect_to=page&wcm_redirect_id=250.

